# The diagnosis of focal liver lesions in pediatric patients

**DOI:** 10.1590/0100-3984.2020.53.3e3

**Published:** 2020

**Authors:** Rubens Chojniak

**Affiliations:** 1 Department of Imaging, A.C.Camargo Cancer Center, São Paulo, SP, Brazil. Email: chojniak@accamargo.org.br.

In the pediatric population, focal liver lesions can have a variety of causes The differential diagnoses of such lesions mainly include congenital cysts, abscesses, and benign or malignant neoplasms^(1,2)^.

Congenital hepatic cysts are rare, typically diagnosed incidentally and usually asymptomatic. The management of a congenital hepatic cyst is typically conservative with periodic monitoring by ultrasound, especially for large cysts. Most hepatic cysts resolve spontaneously, surgical intervention with aspiration, sclerotherapy, or excision being indicated only for cases in which there is hemorrhage, torsion, a progressive increase in size, or imaging characteristics that lead to diagnostic uncertainty^(3)^.

Like congenital hepatic cysts, liver abscess is uncommon in the pediatric population. However, it is a serious infectious disease that is responsible for hospital admissions and even deaths worldwide. Although most abscesses are caused by bacterial infection, cases of infection by protozoa (amebiasis) or fungi (candidiasis) can occur. The diagnosis of liver abscess is usually made on the basis of clinical and laboratory data, together with the presence of focal liver lesions showing characteristics consistent with an abscess. The standard treatment is antibiotic therapy, with or without percutaneous drainage. Most patients with liver abscess have a favorable evolution, although death from liver abscess occurs in most case series studies^(4)^.

In the pediatric population, liver neoplasms can be malignant or benign. The most common malignant liver neoplasm is a metastasis. Primary liver malignant neoplasms account for a small proportion of solid tumors that occur in the pediatric population; hepatoblastoma and hepatocellular carcinoma are the most common such neoplasms, together accounting for approximately two thirds of all primary malignant pediatric liver neoplasms^(1,2)^.

The most common primary malignant liver tumor in the pediatric population is hepatoblastoma, the cells of which resemble those of the embryonic liver. Most cases appear in the first two years of life, and there are few cases that are congenital or are diagnosed after the age of five years. A slight predominance of occurrence in boys has been reported. The typical presentation is of an abdominal mass, together with anemia and vomiting, in a child under three years of age. Serum levels of alpha-fetoprotein (AFP) are high in most cases, and the staging is based on the location of the tumor, which directly correlates with patient survival. Complete surgical resection continues to be the standard treatment for hepatoblastoma, either as the initial treatment or after neoadjuvant chemotherapy^(5,6)^.

Hepatocellular carcinoma is a less common liver malignancy than is hepatoblastoma, accounting for approximately one quarter of pediatric liver neoplasms, and its incidence peaks during two periods of life: between 0 and 4 years; and between 10 and 14 years. Predisposing conditions include liver fibrosis/cirrhosis secondary to metabolic liver disease, viral hepatitis, extrahepatic biliary atresia, total parenteral nutrition, and chemotherapy-induced fibrosis.

Serum AFP is elevated in approximately half of all cases of hepatocellular carcinoma. Metastases usually occur in the lung and lymph nodes. The staging system is similar to that used in hepatoblastoma. More than 70% of these tumors are considered unresectable at the time of presentation, and, unlike hepatoblastomas, hepatocellular carcinomas do not usually respond well to chemotherapy. In most cases, the only potentially curative treatment options are complete surgical resection and transplantation. Recently developed therapeutic strategies include chemoembolization, intra-arterial chemotherapy, and intraoperative cryotherapy. The overall survival rate is low, and pediatric patients with initially resectable disease have a much better prognosis than do those with locally advanced or metastatic disease^(6,7)^.

Other primary malignant tumors of the liver include undifferentiated sarcoma, rhabdomyosarcoma of the biliary tract, angiosarcoma, and rhabdoid tumors^(1,2)^.

In the pediatric population, approximately one third of primary liver neoplasms are benign. As demonstrated in previous studies^1,2)^, the most common benign liver tumors are hemangiomas, hamartomas, and focal nodular hyperplasia (FNH).

Hemangiomas are the most common benign liver tumors in the pediatric population, typically occurring in fetuses, neonates, or infants ≤ 6 months of age. They feature endothelial cells lining the vascular spaces and range from small masses, identified as incidental findings, to large cavernous hemangiomas that are distinguished by large vascular spaces and low cellularity. The natural history of a hemangioma is spontaneous regression within the first two years of life; however, treatment may be necessary when there are symptoms or complications such as heart failure^(2,8)^.

Mesenchymal hamartomas are uncommon benign pediatric liver tumors. They are observed mainly in children under two years of age, and some authors consider them developmental abnormalities rather than neoplasms. Like other hamartomas, mesenchymal hamartomas of the liver are disorganized lesions that are usually cystic and contain varying amounts of soft tissue. They typically arise during the first few months of life, after which they stabilize, continue to grow, or regress. Mesenchymal hamartomas are generally asymptomatic and have a good prognosis; the treatment options are resection and observation^(2,8,9)^.

Hepatic adenoma and FNH are rarely diagnosed in childhood. Both are benign lesions, are associated with a high estrogen environment, and occur predominantly in adolescent girls. Hepatic adenomas are associated with the use of oral contraceptives. Signs and symptoms may be absent or are nonspecific, the latter including abdominal pain and a liver mass. FNH has no malignant potential, is generally asymptomatic, and can be monitored by serial ultrasound whereas symptomatic or rapidly growing lesions can be treated by resection or embolization. Larger hepatic adenomas can be treated with complete surgical excision, because they present a low risk of rupture or hemorrhage^(2,8)^.

Most pediatric patients with focal liver masses are investigated because they present with abdominal pain or abnormal physical examination findings, such as an abdominal mass or abdominal distension. The diagnosis of a pediatric liver tumor is based on the clinical characteristics, serum AFP level, patient age, and imaging characteristics. Ultrasound is the initial imaging modality of choice, because it does not emit ionizing radiation and does not require sedation, as well as because it can detect, characterize, and provide information on the extent of focal liver lesions^(1,2,6,8,10)^.

In a pictorial essay published in the previous issue of Radiologia Brasileira, Rocha et al.^(11)^ presented a variety of pediatric focal liver lesions, which can have a hyperechoic aspect on ultrasound. They can result from trauma or fatty liver disease; they can also be congenital or neoplastic (benign or malignant) lesions. Color Doppler ultrasound can suggest the vascular nature of a tumor, such as infantile hemangioma and FNH.

Although a liver mass can sometimes present a characteristic appearance on ultrasound, subsequent CT or MRI scans are often performed for better characterization, precise evaluation of the extent of the mass, and detection of any associated metastatic disease in cases of a malignant liver neoplasm. When CT is used, the radiation risks can be minimized with the dose reduction techniques outlined in the guidelines published by the Image Gently^(12)^. If a contrasted multiphase study is indicated, MRI can be considered. Although MRI does not expose patients to radiation, sedation is generally required for younger patients. Despite all these resources, there are many cases in which only biopsy findings can provide the definitive diagnosis of a liver mass identified in childhood^(11)^.

In conclusion, knowledge of the pathological spectrum, epidemiological characteristics, and imaging aspects of liver lesions in the pediatric population is essential. Such knowledge guides radiologists in assessing and providing appropriate treatment to pediatric patients with focal liver lesions, thus minimizing the risks of the investigation.
